# Secondary Traumatic Stress Among Emergency Medical Personnel: A Cross-Sectional Study in Romania

**DOI:** 10.3390/nursrep16030102

**Published:** 2026-03-19

**Authors:** Claudia Raluca Balasa Virzob, Florin Gabriel Crisan, Camelia Melania Fizedean, Norberth-Istvan Varga, Mircea Iurciuc, Adelina-Marioara Gherman, Stela Iurciuc

**Affiliations:** 1 Department of Nursing, “Victor Babes” University of Medicine and Pharmacy, Eftimie Murgu Square, No. 2, 300041 Timisoara, Romania; virzob.claudia@umft.ro (C.R.B.V.); fizedean.camelia@umft.ro (C.M.F.); norberth.varga@umft.ro (N.-I.V.); iurciuc.mircea@umft.ro (M.I.); iurciuc.stela@umft.ro (S.I.); 2 Doctoral School, “Victor Babes” University of Medicine and Pharmacy, Eftimie Murgu Square, No. 2, 300041 Timisoara, Romania; florin.crisan@umft.ro

**Keywords:** secondary traumatic stress scale, secondary traumatic stress, emergency medical services, emergency medicine, ambulance, emergency department

## Abstract

**Background/Objectives**: Secondary traumatic stress (STS) affects healthcare professionals indirectly exposed to patients’ trauma, and emergency personnel may be particularly vulnerable. Evidence from Romania is limited. **Methods**: We conducted a cross-sectional survey (July–August 2025) among emergency medical professionals working across the integrated emergency care system in Timisoara, Romania (prehospital ambulance/SMURD services and hospital Emergency Department). Secondary Traumatic Stress (STS) symptoms were measured using the 17-item Secondary Traumatic Stress Scale (STSS; item coding 1–5). We summarized STSS total/subscale scores and reliability, classified total scores into severity categories (0–68 metric), compared scores by workplace, sex, and professional role, and examined associations with age and years of experience. **Results**: The analytic sample included 145 participants (49.0% women), with a median age of 44 years [33–50] and median professional experience of 10 years [5–15]. Mean total STSS was 36.4 (SD 11.9; range 17–66) and internal consistency was high (Cronbach’s alpha = 0.92). Most participants were classified as little/no STS (77.2%), followed by mild (12.4%), moderate (5.5%), high (4.1%), and severe (0.7%). STSS scores did not differ significantly between ambulance service and ED/UPU staff. Women reported higher total STSS than men (39.0 vs. 33.9; *p* = 0.010), with significant differences for intrusion (*p* = 0.035) and arousal (*p* = 0.004). Role differences were significant for total STSS, intrusion, and arousal (*p* ≤ 0.031), with nurses scoring higher than ambulance drivers/attendants in post hoc comparisons. Years of experience showed small positive correlations with total STSS (r = 0.18, *p* = 0.032) and intrusion (r = 0.21, *p* = 0.010); age was associated with intrusion only (r = 0.22, *p* = 0.008). **Conclusions**: In this Romanian emergency care cohort, most participants reported low STS severity, but a clinically relevant minority had moderate-to-severe symptoms. Higher symptom burden among women and nurses suggests groups that may benefit from targeted monitoring and support within the integrated emergency system.

## 1. Introduction

Secondary traumatic stress (STS) denotes a constellation of intrusion, avoidance and arousal symptoms experienced by helpers who are indirectly exposed to traumatic events [1,2]. It is distinct from burnout and depression but correlates strongly with them; a recent meta-analysis across healthcare professionals reported a weighted correlation coefficient of 0.63 between STS and burnout during the COVID-19 pandemic [1]. Unchecked STS can impair professionals’ mental health, diminish empathy and job satisfaction, and contribute to staff turnover, thereby threatening the quality of patient care and the stability of emergency services [3].

Evidence consistently shows that professionals working in emergency and prehospital settings are particularly vulnerable to STS. Cross-sectional surveys of emergency department nurses reveal that 64–85% report at least one STS symptom [4,5], and up to a third meet diagnostic criteria [5]. In a statewide survey of U.S. EMS personnel, 40.9% experienced vicarious trauma and 24% had contemplated suicide [6]. According to a systematic review, around 65% of emergency nurses worldwide experience secondary traumatic stress [7]. Factors such as frequent exposure to critical incidents, high patient acuity, and moral dilemmas may amplify STS risk in these settings. The prevalence of STS appears higher in Emergency Department (ED) nurses than in other hospital units, and paramedics and ambulance drivers often face similar hazards [2,5].

Research has identified multiple correlates of STS in emergency personnel, but findings vary across studies. Female sex, lower resilience, personal trauma history and high proportion of trauma-related tasks were associated with higher STS scores in a multi-centre survey of emergency physicians and advanced practice providers [8]. Years of service, burnout, and childhood emotional neglect predicted vicarious trauma and suicidality in another EMS cohort [6]. In Saudi Arabia, female nurses, older age and longer experience were linked to higher STSS scores [9], whereas a Polish study found that lower job satisfaction and maladaptive cognitive coping strategies predicted STS symptoms [2]. Cross-shift analyses during the COVID-19 pandemic showed that midshift nurses had higher burnout and STS levels than day-shift nurses [10]. These inconsistencies underscore the contextual nature of STS and highlight the importance of examining predictors within specific health systems and cultures.

Emergency medical services in Romania integrate prehospital (Ambulance Service and SMURD—“Serviciul Mobil de Urgență, Reanimare și Descarcerare”) with hospital-based emergency (UPU—“Unitatea de Primiri Urgențe”) departments. These settings are characterised by limited resources, high case volumes and frequent exposure to severe trauma. Yet, to our knowledge, no published study has quantified STS levels among Romanian emergency professionals using a validated instrument such as the Secondary Traumatic Stress Scale (STSS). Without local data, it is impossible to determine whether international findings apply to Romania or to develop targeted interventions. Given the distinctive structure of the Romanian system, including SMURD teams embedded within UPU and overlapping roles for nurses, physicians and ambulance staff, understanding STS in this context is both clinically and organisationally imperative.

This study addresses the gap by assessing secondary traumatic stress among emergency medical professionals in Western Romania. Using a Romanian-language administration of the 17-item Secondary Traumatic Stress Scale, we aimed to (i) describe total and subscale STS scores and estimate the reliability of the instrument; (ii) determine the distribution of participants across established severity categories; (iii) compare STS levels across professional roles (nurses, physicians, ambulance drivers/paramedics) and workplace settings (prehospital vs. UPU/SMURD); and (iv) explore associations between STS scores and demographic/occupational variables such as sex, age and years of professional experience. Findings from this study will inform occupational health strategies and lay the groundwork for interventions to mitigate secondary traumatic stress among emergency medical personnel in Romania.

## 2. Materials and Methods

### 2.1. Study Design and Setting

This study was designed as a cross-sectional observational survey of secondary traumatic stress symptoms, assessed via the Secondary Traumatic Stress Scale (STSS) questionnaire, among emergency medical personnel working in Timisoara, a Romanian city of approximately 300,000 inhabitants. Data collection took place between 20 July and 15 August 2025. The survey targeted staff working in both prehospital (ambulance and “SMURD”) and hospital-based (emergency department) emergency care settings.

In Romania, SMURD (“Serviciul Mobil de Urgență, Reanimare și Descarcerare”) refers to the mobile emergency service providing prehospital emergency response, resuscitation support, and extrication. Participants in the present study were recruited from the Timis County Ambulance Service (including SMURD components where applicable) and from the Emergency Department of the Municipal Emergency Hospital of Timisoara, Romania.

### 2.2. Participants

Eligible participants were physicians, nurses, ambulance drivers/attendants, and paramedics actively working in the Ambulance Service of Timis County, Romania, and the Emergency Department of the Municipal Emergency Hospital of Timisoara, Romania. Inclusion criteria were: employment in an emergency care role at the time of the survey and the ability to complete a Romanian-language questionnaire. No additional inclusion criteria were mandatory. Individuals were excluded if they provided incomplete STSS item responses or did not consent to participate. At the time of data collection, 151 eligible emergency care staff members were employed across the two participating institutions.

Workplace was recorded using predefined response options (e.g., ambulance, SMURD, ED). During statistical analysis, SMURD and ambulance staff were merged into the same category, due to the similar nature of their medical duties (prehospital medical care).

### 2.3. Data Collection

Data were collected using the Secondary Traumatic Stress Scale (STSS) questionnaire [10], in Romanian. The questionnaire invitation was distributed to the full eligible emergency care workforce of the two participating institutions via institutional email lists, internal messaging groups, and in person, and responses were collected using paper forms. Participation was voluntary and anonymous; no directly identifying personal information was collected. Prior to completion, participants (ED doctors and nurses, ambulance drivers, etc.) were presented with proof of Ethical Approval by the Ethics Committee of the County Ambulance Service of Timis County. Survey responses were exported to a spreadsheet for analysis.

### 2.4. Measures

#### 2.4.1. Sociodemographic and Occupational Variables

The questionnaire collected basic sociodemographic and occupational characteristics, including age, sex, professional role (physician, nurse, ambulance driver/attendant, or paramedic), primary workplace setting (prehospital ambulance service versus ED), years of professional experience, and work schedule (e.g., shift work versus day schedule). Age was recorded as a numeric value and then, for descriptive reporting, age was also presented in grouped form (<35, 35–44, 45–54, and ≥55 years).

#### 2.4.2. Secondary Traumatic Stress Scale (STSS)

Secondary traumatic stress symptoms were assessed using the Secondary Traumatic Stress Scale (STSS), a 17-item instrument designed to measure intrusion, avoidance, and arousal symptoms associated with indirect exposure to traumatic events [11]. In the original validation study, the STSS showed strong internal consistency for the total scale and subscales (total α = 0.93; Intrusion α = 0.80; Avoidance α = 0.87; Arousal α = 0.83), together with evidence of convergent, discriminant, and factorial validity [11]. In the present study, the instrument was administered in Romanian. To our knowledge, a peer-reviewed Romanian psychometric validation study reporting formal reliability and factor-analytic evaluation is not currently available; accordingly, the instrument used here should be understood as a Romanian-language administration of the standard 17-item STSS rather than a separately re-validated national adaptation. Participants were instructed to rate how frequently each statement applied to their experiences in the previous week in the context of providing care for critically ill or severely injured patients. Each item was presented on a five-point Likert scale from 1 (“Niciodată”/never) to 5 (“Foarte frecvent”/very frequently).

A total STSS score was computed as the sum of the 17 item scores (possible range: 17–85). Subscale scores were computed as sums of their respective items using the standard STSS item structure: Intrusion (items 2, 3, 6, 10, 13), Avoidance (items 1, 5, 7, 9, 12, 14, 17), and Arousal (items 4, 8, 11, 15, 16) [11]. Higher scores indicate greater frequency of secondary traumatic stress symptoms.

#### 2.4.3. STSS Severity Classification

To facilitate prevalence-style reporting alongside continuous score summaries, total STSS scores were also classified using published severity thresholds derived for the original STSS scoring framework [10]. The Romanian-language questionnaire administered in this study retained the standard 17 STSS items and response options, but raw responses were recorded on a 1–5 scale, yielding total scores from 17 to 85. Because the published severity thresholds are defined on the original 0–4 per-item metric (total range: 0–68), raw total scores were converted to that metric by subtracting 1 point from each item (equivalently, subtracting 17 points from the 17–85 total score) before applying the thresholds. This conversion was performed solely to align severity-category reporting with the published STSS cutoffs and does not represent a different Romanian scoring system or a modification of the instrument itself.

Severity categories were defined as: little/none (≤27), mild (28–37), moderate (38–43), high (44–48), and severe (≥49) on the 0–68 total score scale. Continuous analyses in the manuscript used the observed 17–85 total scores and corresponding subscale sums; the 0–68 transformation was used only for categorical severity reporting.

### 2.5. Statistical Analysis

Statistical analyses were performed using SPSS version 27 (IBM Corp., Armonk, NY, USA). Continuous variables are described using means and standard deviations and, where informative, medians with interquartile ranges reported in square brackets. Categorical variables are presented as counts and percentages.

Internal consistency of the STSS total scale and subscales was evaluated using Cronbach’s alpha. For two-group comparisons (e.g., prehospital ambulance service versus ED; female versus male), Welch’s *t*-test was used to compare mean scores. Effect sizes for two-group comparisons were quantified using Cohen’s d. Mean differences are reported with 95% confidence intervals, and all statistical tests were two-sided with a significance threshold of alpha = 0.05.

For comparisons across professional roles, one-way analysis of variance (ANOVA) was conducted for the total STSS score and subscale scores. Eta-squared (η^2^) was reported as an effect size measure for ANOVA. When the overall ANOVA was statistically significant, pairwise post hoc comparisons were performed using Tukey’s honestly significant difference (HSD) procedure. Associations between STSS scores and years of professional experience and age were evaluated using Pearson correlation coefficients, with 95% confidence intervals derived using Fisher’s z transformation. Workplace analyses were focused on the two main groups (ambulance service and ED).

### 2.6. Ethical Considerations

The study protocol was reviewed and approved by the Ethics Committee of the Timis County Ambulance Service (approval number 7350 from 16 July 2025), and later approved for publication by the Ethics Committee of the Municipal Emergency Hospital of Timisoara (approval number E-177/19.01.2026, from 19 January 2026). All procedures were conducted in accordance with applicable national regulations and the principles of the Declaration of Helsinki. Participation was voluntary, and informed consent was obtained from all participants prior to questionnaire completion. Data were collected and analyzed in anonymized form, and results are reported in aggregate to protect participant confidentiality.

## 3. Results

### 3.1. Cohort Description

A total of 151 eligible emergency care staff members from the two participating institutions completed the questionnaire, corresponding to the full working staff at the time of data collection. Six questionnaires with incomplete STSS responses were excluded, yielding a final analytic cohort of 145 participants.

The cohort was nearly evenly distributed by sex, with 71 women (49.0%) and 74 men (51.0%). Age ranged widely, with a mean age of 41.7 years and a median of 44 years [33–50], indicating a predominantly mid-career workforce. Professional experience was substantial overall, with a median of 10 years in emergency medical practice [5–15], although experience levels varied considerably across individuals.

In terms of professional role, nearly half of the participants were nurses (n = 71), followed by physicians (n = 40), ambulance drivers or attendants (n = 25), and a smaller group of paramedics (n = 9). Regarding workplace setting, the majority of respondents were employed in prehospital emergency services (ambulance), while over one third worked primarily in hospital emergency departments (ED).

Most participants reported working rotating shifts, reflecting the operational realities of emergency medical services in Romania. Overall, the cohort represents a heterogeneous and clinically relevant census-based sample of emergency healthcare professionals with substantial exposure to acute, high-stress clinical environments.

Participant characteristics are presented in Table 1.

### 3.2. STSS Scores and Internal Consistency

Across the entire cohort, the mean total STSS score was 36.4 (SD = 11.9), indicating a moderate overall level of secondary traumatic stress symptoms. At the subscale level, mean scores were 12.2 (SD = 4.4) for Intrusion, 13.1 (SD = 4.8) for Avoidance, and 11.1 (SD = 4.2) for Arousal. Descriptive statistics for total and subscale scores are summarised in Table 2.

The internal consistency of the STSS in this sample was high. Cronbach’s alpha for the total scale was 0.92, indicating excellent reliability. Subscale reliability was also satisfactory, with alpha coefficients of 0.86 for Intrusion, 0.80 for Avoidance, and 0.85 for Arousal.

### 3.3. STSS Severity Categories

When classified according to established STSS severity thresholds, the majority of participants fell within the little or no secondary traumatic stress category. A smaller proportion of the cohort exhibited mild symptoms, while progressively fewer individuals met criteria for moderate, high, or severe levels of secondary traumatic stress.

Overall, approximately one quarter of participants reported symptom levels exceeding the minimal range, and around 10% met criteria for moderate to severe secondary traumatic stress. The full distribution of participants across severity categories is presented in Table 3.

### 3.4. Comparison by Workplace and Sex

Workplace comparisons focused on the two primary settings in the dataset: the prehospital ambulance service (including ambulance and SMURD medical staff) and the hospital Emergency Department. No statistically significant differences were observed between ambulance service and ED staff for the total STSS score or any subscale (Table 4).

Female participants reported higher total STSS scores than male participants (mean 39.0 vs. 33.9; *p* = 0.010). Differences were most pronounced for the Arousal subscale (mean 12.1 vs. 10.1; *p* = 0.004), while Avoidance showed a similar direction but did not reach statistical significance at the 0.05 level (Table 5).

### 3.5. Comparison by Professional Role

Mean STSS scores differed across professional roles (Table 6). Nurses reported the highest average symptom burden, with a mean total STSS score of 38.8 ± 12.6, compared with 36.4 ± 9.5 among physicians, 31.3 ± 10.8 among ambulance drivers/attendants, and 31.8 ± 15.0 among paramedics. A similar pattern was observed for the Intrusion and Arousal subscales: nurses had higher mean Intrusion (13.4 ± 4.5) and Arousal (11.8 ± 4.4) scores than ambulance drivers/attendants (Intrusion 10.3 ± 4.3; Arousal 9.2 ± 3.6) and paramedics (Intrusion 11.8 ± 5.6; Arousal 9.0 ± 5.0), while physicians showed intermediate values (Intrusion 11.2 ± 3.5; Arousal 11.5 ± 3.6). These role-specific descriptive patterns should be interpreted cautiously because subgroup sizes were unequal, particularly for the paramedic group (n = 9), which limits estimate precision and reduces power for between-role comparisons.

Overall role-related differences were statistically significant for total STSS (F(3, 141) = 3.05, *p* = 0.031, η^2^ = 0.06), Intrusion (F(3, 141) = 4.16, *p* = 0.007, η^2^ = 0.08), and Arousal (F(3, 141) = 3.45, *p* = 0.018, η^2^ = 0.07), indicating small-to-moderate effects. In contrast, Avoidance did not differ significantly by role (F(3, 141) = 1.61, *p* = 0.189, η^2^ = 0.03). Post hoc Tukey testing identified one consistent pairwise difference: nurses scored higher than ambulance drivers/attendants for total STSS (Δ = 7.48), Intrusion (Δ = 3.10), and Arousal (Δ = 2.63), whereas other role comparisons did not reach statistical significance (Table 7).

### 3.6. Associations with Professional Experience and Age

Years of professional experience showed a weak positive association with overall secondary traumatic stress symptom burden (Table 8). Total STSS increased modestly with experience (r = 0.18, 95% CI 0.02 to 0.33, *p* = 0.032), and the association was slightly stronger for Intrusion (r = 0.21, 95% CI 0.05 to 0.36, *p* = 0.010). In contrast, correlations between experience and Avoidance (r = 0.13, *p* = 0.132) and between experience and Arousal (r = 0.14, *p* = 0.100) did not reach statistical significance.

Age was not significantly associated with total STSS (r = 0.12, *p* = 0.144). However, age showed a small positive association with the Intrusion subscale (r = 0.22, 95% CI 0.06 to 0.37, *p* = 0.008), while associations with Avoidance (r = 0.03, *p* = 0.742) and Arousal (r = 0.08, *p* = 0.327) were not statistically significant (Table 8).

## 4. Discussion

### 4.1. Summary of Main Findings

In this cross-sectional cohort of 145 emergency medical professionals from Western Romania, we captured secondary traumatic stress symptoms across the integrated emergency care system (prehospital ambulance services and the hospital Emergency Department/UPU, including SMURD personnel). Overall secondary traumatic stress symptom burden was moderate on average, but highly variable between individuals. The mean total STSS score was 36.4 (SD 11.9), spanning a broad range (17–66), indicating that while some participants reported little symptomatology, others reported frequent symptoms across multiple items. At the symptom-domain level, avoidance tended to be the most prominent domain in the cohort, while intrusion and arousal were also clearly represented.

The STSS also showed strong psychometric performance in this sample. Internal consistency was excellent for the total scale (Cronbach’s α = 0.92) and good for the subscales (α = 0.80–0.86), supporting the reliability of the score patterns reported in the subgroup analyses.

Comparisons between the two main work settings—prehospital ambulance service and hospital ED/UPU—did not show statistically significant differences in total STSS or any subscale, suggesting broadly similar symptom burden across these settings within the same integrated emergency system. In other words, secondary traumatic stress in this cohort was not concentrated in one workplace setting.

In contrast, sex-based comparisons identified a consistent pattern of higher symptom burden among women. Women reported significantly higher total STSS scores than men (*p* = 0.010), with statistically significant differences in intrusion (*p* = 0.035) and especially arousal (*p* = 0.004). Avoidance also trended higher among women but did not reach statistical significance (*p* = 0.063). This pattern suggests that sex differences in this cohort were driven more by intrusive re-experiencing and physiological/psychological arousal symptoms than by avoidance behaviors alone.

Professional role comparisons further indicated that symptom burden was not uniformly distributed across occupational groups. Overall differences across roles were statistically significant for total STSS (*p* = 0.031), intrusion (*p* = 0.007), and arousal (*p* = 0.018), but not for avoidance (*p* = 0.189). Nurses showed the highest mean symptom levels, and the only consistent post hoc difference was between nurses and ambulance drivers/attendants for total score, intrusion, and arousal. This implies that role-related variation in secondary traumatic stress in this cohort was again concentrated in intrusion and arousal domains, rather than avoidance.

Finally, correlational analyses suggested modest relationships between symptom burden and professional time-in-service. Years of experience showed a small but statistically significant positive association with total STSS (r = 0.18, *p* = 0.032) and intrusion (r = 0.21, *p* = 0.010), while associations with avoidance and arousal did not reach statistical significance. Age was not significantly associated with total STSS, but showed a small significant association with intrusion (r = 0.22, *p* = 0.008). Taken together, these findings suggest that intrusion symptoms—more than avoidance or arousal—were the symptom domain most consistently linked to cumulative time-related variables in this cohort.

These findings matter because traumatic stress in emergency nurses has been linked, in prior evidence, to consequences that extend beyond individual distress, including absenteeism/turnover and potential impacts on care quality and organisational commitment [3].

### 4.2. Comparison with Previous Reports

When compared with previously published data, the severity distribution observed in our cohort appears lower than that reported in many emergency nursing samples, particularly those assessed during the COVID-19 period. A recent systematic review and meta-analysis of emergency nurses reported a pooled secondary traumatic stress prevalence of 65%, with regional estimates of 74% in Asia, 59% in North America, and 53% in Europe; studies conducted during the pandemic showed an even higher pooled prevalence of 70% (95% CI 62–78%) [7]. In line with these estimates, a cross-sectional study conducted in three emergency departments in Ireland reported that 64% (67/105) of emergency nurses met STSS criteria for secondary traumatic stress [4]. In a cross-sectional survey of emergency nurses in Jordan (n = 202), 94% of participants scored ≥28 on the STSS, indicating secondary traumatic stress of varying severity, with a mean total STSS score of 46 (SD 12.45). Severity categorization in that study showed a large proportion of respondents classified as severe (40%), alongside 12.3% high and 22.2% moderate STS [12]. Together, these studies illustrate that both prevalence and severity distributions of STS among emergency nurses can exceed those observed in the present cohort.

Beyond individual prevalence estimates, recent reviews emphasize marked heterogeneity in how secondary traumatic stress is conceptualized and measured across emergency care studies. A recent scoping review noted that some studies treat secondary traumatic stress as a distinct construct, whereas others embed it within broader compassion fatigue frameworks [13]. Likewise, an integrative review of occupational distress in emergency nurses highlighted that distress-related outcomes are shaped by interacting individual, organizational, and role-related factors [14]. This heterogeneity complicates direct numerical comparisons between studies.

Differences across studies are also reflected at the symptom-domain level. A study from Marković et al. [15], examining coping styles and STSS symptom clusters, suggested that variation in intrusion, avoidance, and arousal subscale scores across cohorts may reflect differences in coping patterns, not just exposure. An integrative review of resilience in nursing described resilience as a dynamic adaptation process and reported that higher resilience is generally associated with more favorable mental health and work-related outcomes (e.g., lower burnout/depression and higher job/life satisfaction), and that resilience-building interventions can improve coping self-efficacy and emotion regulation [16]. In the context of our comparatively lower moderate-to-severe STS proportion, this literature supports interpreting prevalence and severity distributions across cohorts with caution, because unmeasured resilience-related resources may contribute to between-study differences even when the same instrument is used [16].

Pandemic-period investigations consistently report elevated STSS scores relative to non-pandemic samples. For example, a Greek nursing cohort assessed during COVID-19 had a mean total STSS score of 44.00 [17], and an Italian survey of medical staff and emergency workers reported similarly high mean scores of approximately 40.5 [18]. Large international data collected during the outbreak also showed substantial secondary traumatic stress prevalence, especially among frontline personnel and those exposed to patients’ death [19]. These findings support the view that pandemic-era studies are likely to have captured unusually high levels of symptom burden.

Outside emergency medicine, secondary traumatic stress remains prevalent but is generally reported at lower levels. In a psychological risk assessment conducted among nurses in a German university hospital, 25.3% reported secondary traumatic symptoms [20], while a study of Australian alcohol and other drug workers found that 19.9% met STSS criteria [21]. Although these populations differ from emergency medical personnel, they provide additional context for interpreting the range of STS prevalence observed across helping professions.

Several factors may help explain why the severity distribution in our cohort was lower than that reported in many published studies. First, our sample was not restricted to emergency nurses, but also included physicians, ambulance drivers/attendants, and paramedics; in our own data, nurses showed the highest mean STSS scores, whereas ambulance drivers/attendants and paramedics had lower mean values, so a mixed occupational sample would be expected to yield a lower overall severity distribution than nurse-only cohorts. Second, our study was conducted in a non-pandemic period, whereas several of the highest published estimates were obtained during the COVID-19 period, when workload, mortality exposure, and organizational strain were unusually intense. Third, the present study reflects one local integrated Romanian emergency care system combining prehospital and hospital emergency services, and this organisational context may shape trauma exposure patterns, teamwork, and informal peer support differently from the settings examined elsewhere. Finally, because our cohort was voluntary and single-region, we cannot exclude selection effects or unmeasured protective factors—such as resilience, coping style, supervisory support, or local organizational culture—that may have contributed to lower observed symptom severity. For these reasons, the lower severity distribution observed here is best interpreted as likely reflecting differences in sample composition and context rather than evidence that secondary traumatic stress is uniformly lower across Romanian emergency personnel.

### 4.3. Practical Implications

From a practical standpoint, these findings support routine monitoring of secondary traumatic stress symptoms within Romanian emergency services, particularly among nurses and staff with longer professional exposure. Occupational health programmes could incorporate brief periodic screening, structured peer-support or reflective debriefing opportunities, and access to confidential psychological support for personnel reporting persistent intrusion or arousal symptoms. Because symptom burden did not differ materially between prehospital and hospital emergency settings within this integrated system, preventive strategies should be implemented across the emergency care pathway rather than restricted to a single workplace.

### 4.4. Limitations

Several limitations should be considered when interpreting our results. First, the cross-sectional design precludes causal inference and does not capture symptom trajectories. Second, the cohort should be interpreted as a convenience sample rather than as a representative sample of Romanian emergency medical personnel. Participants were recruited from one county-level integrated emergency system (Timisoara) through voluntary participation across two institutions, which may introduce selection and non-response bias and limit generalizability to other Romanian regions or emergency care structures. It is also possible that staff with either lower or higher symptom burden were differentially likely to participate. Third, outcomes were based on self-report over a one-week recall period; this may be influenced by reporting tendencies and does not substitute for clinical assessment. While the STSS has been psychometrically validated in multiple languages and populations internationally, a formal Romanian-language validation (with published reliability and factor analysis data) is not available in the literature. Another limitation is that professional-role comparisons were based on unequal subgroup sizes, especially the small paramedic subgroup (n = 9), which limits the precision and stability of between-role estimates and may have reduced power to detect true differences. Finally, we did not measure key potential modifiers and correlates such as objective trauma exposure intensity, prior trauma history, resilience, burnout, depression, or organizational factors, and analyses were unadjusted for confounding. Future research should include multicenter Romanian samples, incorporate broader occupational and psychological measures, and use longitudinal designs to clarify determinants and to evaluate targeted interventions for mitigating secondary traumatic stress in emergency personnel

## 5. Conclusions

In this cohort of emergency medical professionals working across prehospital ambulance/SMURD services and the hospital ED/UPU in Timisoara, secondary traumatic stress symptoms were, on average, in the moderate range, but most participants fell into the little/none severity category. A smaller subgroup reported moderate-to-severe symptom levels, underscoring that clinically meaningful distress is present within the workforce even when overall prevalence estimates appear lower than in many international emergency-nurse samples. Symptoms did not differ significantly by workplace setting within this integrated system, whereas women and nurses showed higher STSS scores, particularly in intrusion and arousal domains. The modest associations between intrusion symptoms and both professional experience and age are consistent with potential cumulative effects of repeated trauma exposure over time. These findings support routine monitoring of secondary traumatic stress and the development of system-level and role-sensitive support strategies in Romanian emergency services.

## Figures and Tables

**Table 1:** Participant Characteristics.

Characteristic	Value
Total participants, n	145
Sex, n (%)	
Female	71 (49.0)
Male	74 (51.0)
Age (years), median [IQR]	44 [33–50]
Age group, n (%)	
<35	44 (30.3)
35–44	29 (20.0)
45–54	61 (42.1)
≥55	11 (7.6)
Professional role, n (%)	
Nurse	71 (49.0)
Physician	40 (27.6)
Ambulance driver/attendant	25 (17.2)
Paramedic	9 (6.2)
Workplace, n (%)	
Ambulance service	89 (61.4)
Emergency Department	52 (35.9)
SMURD	4 (2.8)
Professional experience (years), median [IQR]	10 [5–15]
Work schedule, n (%)	
Shift work	139 (95.9)
Day schedule	5 (3.4)
Other schedule	1 (0.7)

**Table 2:** STSS descriptive statistics and reliability. STSS = Secondary Traumatic Stress Scale.

Measure	Mean ± SD	Min–Max	Cronbach’s α
Total STSS (17 items; range 17–85)	36.4 ± 11.9	17–66	0.92
Intrusion (5 items; range 5–25)	12.2 ± 4.4	5–23	0.86
Avoidance (7 items; range 7–35)	13.1 ± 4.8	7–27	0.80
Arousal (5 items; range 5–25)	11.1 ± 4.2	5–21	0.85

**Table 3:** STSS severity categories based on linearly transformed total scores (0–68 metric).

Severity Category (0–68 Scale)	n	%
Little/none (≤27)	112	77.2
Mild (28–37)	18	12.4
Moderate (38–43)	8	5.5
High (44–48)	6	4.1
Severe (≥49)	1	0.7

**Table 4:** STSS comparison by workplace (Welch *t*-tests). ED = Emergency Department; CI = Confidence Interval.

Outcome	Ambulance Mean ± SD	ED Mean ± SD	Mean Diff	95% CI	*p*
Total STSS	35.8 ± 12.1	36.7 ± 11.6	−0.92	−4.99 to 3.15	0.655
Intrusion	12.0 ± 4.5	12.4 ± 4.4	−0.43	−1.96 to 1.11	0.584
Avoidance	13.0 ± 4.7	13.0 ± 4.8	−0.03	−1.67 to 1.62	0.975
Arousal	10.8 ± 4.3	11.3 ± 4.0	−0.47	−1.89 to 0.95	0.516

**Table 5:** STSS comparison by sex (Welch *t*-tests).

Outcome	Female Mean (SD)	Male Mean (SD)	Mean Diff	95% CI	*p*
Total STSS	39.0 ± 11.6	33.9 ± 11.8	5.04	1.21 to 8.87	0.010
Intrusion	12.9 ± 4.4	11.4 ± 4.3	1.55	0.11 to 2.99	0.035
Avoidance	13.9 ± 4.6	12.4 ± 4.9	1.48	−0.08 to 3.05	0.063
Arousal	12.1 ± 4.2	10.1 ± 3.9	2.01	0.66 to 3.35	0.004

**Table 6:** STSS scores by professional role.

Role	n	Total STSS Mean ± SD	Intrusion Mean ± SD	Avoidance Mean ± SD	Arousal Mean ± SD
Nurse	71	38.8 ± 12.6	13.4 ± 4.5	13.5 ± 5.0	11.8 ± 4.4
Physician	40	36.4 ± 9.5	11.2 ± 3.5	13.7 ± 4.5	11.5 ± 3.6
Ambulance driver/attendant	25	31.3 ± 10.8	10.3 ± 4.3	11.8 ± 4.4	9.2 ± 3.6
Paramedic	9	31.8 ± 15.0	11.8 ± 5.6	11.0 ± 5.2	9.0 ± 5.0

**Table 7:** ANOVA and post hoc results for role comparisons.

Outcome	F(df1, df2)	*p*	η^2^	Significant Tukey Post Hoc Comparisons (*p* < 0.05)
Total STSS	3.05 (3, 141)	0.031	0.06	Ambulance driver vs. Nurse (Δ = 7.48)
Intrusion	4.16 (3, 141)	0.007	0.08	Ambulance driver vs. Nurse (Δ = 3.10)
Avoidance	1.61 (3, 141)	0.189	0.03	None
Arousal	3.45 (3, 141)	0.018	0.07	Ambulance driver vs. Nurse (Δ = 2.63)

**Table 8:** Correlations of STSS scores with years of experience and age.

Outcome	Predictor	r	95% CI	*p*
Total STSS	Experience (years)	0.18	0.02 to 0.33	0.032
Intrusion	Experience (years)	0.21	0.05 to 0.36	0.010
Avoidance	Experience (years)	0.13	−0.04 to 0.28	0.132
Arousal	Experience (years)	0.14	−0.03 to 0.29	0.100
Total STSS	Age	0.12	−0.04 to 0.28	0.144
Intrusion	Age	0.22	0.06 to 0.37	0.008
Avoidance	Age	0.03	−0.14 to 0.19	0.742
Arousal	Age	0.08	−0.08 to 0.24	0.327

## Data Availability

The original contributions presented in this study are included in the article. Further inquiries can be directed to the corresponding author.

## Public Involvement Statement

Participants filled out the STSS questionnaire.

## Guidelines and Standards Statement

This manuscript was drafted against the ‘Strengthening the Reporting of Observational Studies in Epidemiology’ (STROBE) guideline for observational studies.

## Abbreviations

The following abbreviations are used in this manuscript:

STSS	Secondary Traumatic Stress Scale
ED	Emergency Department
